# Local motion detectors are required for the computation of expansion flow-fields

**DOI:** 10.1242/bio.012690

**Published:** 2015-07-31

**Authors:** Tabea Schilling, Alexander Borst

**Affiliations:** Department of Circuits-Computation-Models, Max-Planck-Institute of Neurobiology, Martinsried D-82152, Germany

**Keywords:** Collision avoidance, Looming, Local motion detectors

## Abstract

Avoidance of predators or impending collisions is important for survival. Approaching objects can be mimicked by expanding flow-fields. Tethered flying fruit flies, when confronted with an expansion flow-field, reliably turn away from the pole of expansion when presented laterally, or perform a landing response when presented frontally. Here, we show that the response to an expansion flow-field is independent of the overall luminance change and edge acceleration. As we demonstrate by blocking local motion-sensing neurons T4 and T5, the response depends crucially on the neural computation of appropriately aligned local motion vectors, using the same hardware that also controls the optomotor response to rotational flow-fields.

## INTRODUCTION

Whenever an animal moves or something else is moving in the environment relative to it, visual motion occurs on the retina. Such visual motion cues are of importance particularly for fast flying animals, enabling them to perform various flight maneuvers such as maintaining a straight course, flying towards an object or avoiding it. A well-studied example is the optomotor response, which represents compensatory movements of the body and head syndirectional with rotational large-field motion that may signal deviation from a straight course (Blondeau and Heisenberg, 1982). This behavior is controlled by lobula plate tangential cells as demonstrated by genetic or surgical ablation (Geiger and Nässel, 1981; Hausen and Wehrhahn, 1983; Heisenberg et al., 1978) and activation studies (Haikala et al., 2013). Lobula plate tangential cells receive their input from a 2-dimensional, retinotopically arranged array of columnar T4 and T5 cells (Schnell et al., 2012) with T4 cells responding preferentially to moving bright and T5 to moving dark edges (Maisak et al., 2013).

Other visually controlled behaviors are evoked by expanding optic flow, which is generated on the retina by objects moving towards the fly or by impending collision with stationary objects. Looming stimuli can induce two different behaviors in flying flies dependent on the position of the stimulus. A frontal position of the pole of expansion elicits a landing response (Borst and Bahde, 1988; Braitenberg and Ferretti, 1966), whereas laterally expanding stimuli evoke an avoidance behavior (Tammero and Dickinson, 2002). The avoidance behavior has been studied in freely (Muijres et al., 2014, 2015) as well as in tethered flying flies (Tammero and Dickinson, 2002; Tammero et al., 2004). However, the neuronal basis of both these behaviors is not well understood. We asked whether the T4/T5 cells, which act as local motion detectors known to underlie optomotor responses, are also necessary for avoidance and landing behavior. We first characterized the avoidance and landing response of tethered flying flies using different expanding stimuli. Silencing T4 and T5 neurons genetically, we found that information from local motion circuits is essential for both the avoidance and the landing response. We thus conclude that computation of an expansion flow-field depends on the activity of the same set of elementary motion detecting neurons that control the optomotor response.

## RESULTS AND DISCUSSION

**Fig. 1.**
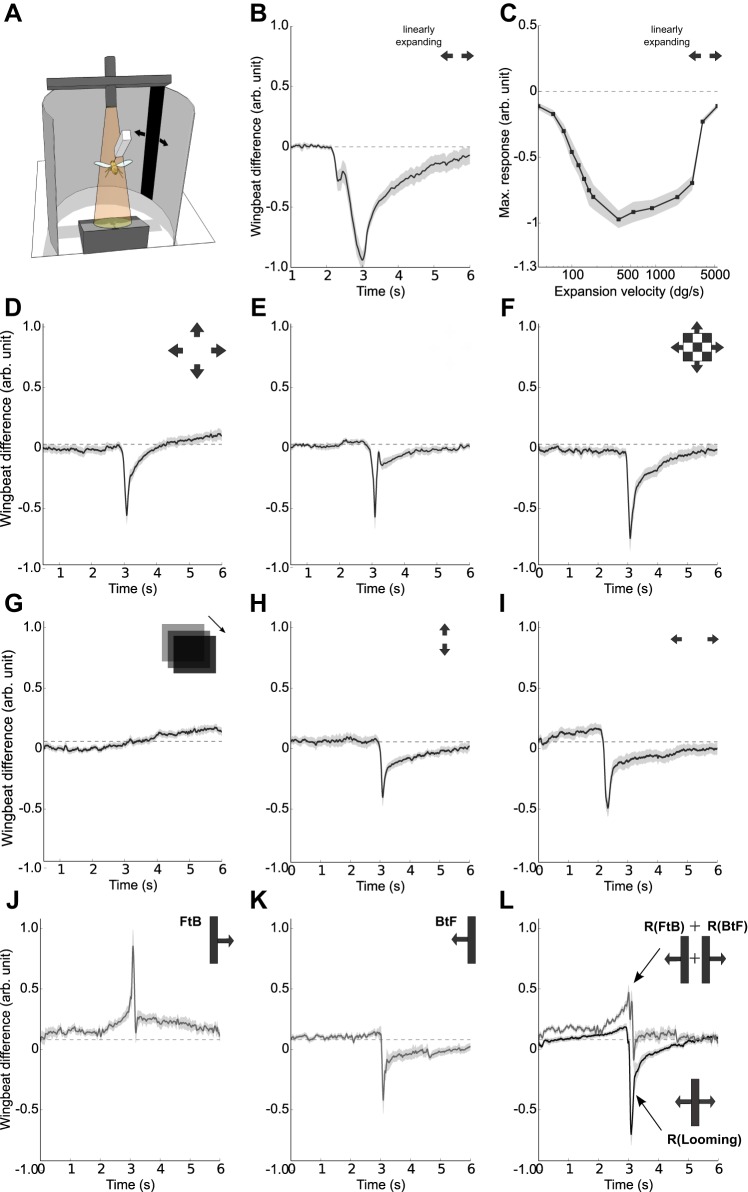
**Characterization of the avoidance behavior elicited by different stimuli.** Average turning responses of Canton-S wild-type flies, elicited by expanding stimuli. (A) Illustration of the flight setup. (B) Avoidance response to a vertical bar expanding horizontally presented at ±50°. The bar expands from 0° to 180° in 1 s, *n*=13. (C) Velocity tuning of the avoidance response to an expanding bar with expansion velocities from 40 to 5400 deg/s. The flies reacted with comparable strong turning to a broad range of expansion velocities from 180° to 2700° with a maximum at 360 deg/s, *n*=10. (D-I) Turning responses to different expansion/looming stimuli, *n*=10. (D-F) Avoidance responses to a dark looming square (D), a bright looming square (E) and a looming square with a checkerboard pattern (F). (G) Response to a dimming 120°×120° square. (H) Avoidance response to a horizontal bar expanding vertically at a velocity of 360 deg/s, width=60°, presented at ±60°. (I) Avoidance of two 10° broad vertical stripes moving away from each other for 0.25 s at a velocity of 360 deg/s. (J,K) Reactions to a looming bar where either the anterior or the posterior edge is moving, *n*=10. (L) The sum of the single edge responses (upper line) and the response to the sum of both edges moving (lower line), *n*=10. FtB, front to back; BtF, back to front. All data represent mean±s.e.m.

In order to characterize visual features which elicit avoidance responses, we confronted tethered flying flies (Fig. 1A) with various visual stimuli presented laterally at an angle of ±50° to the flight course. The first stimulus consisted of a vertical dark bar expanding with different angular velocities to 180° width. A typical collision avoidance response to a bar expanding at a constant velocity of 180 deg/s is shown in Fig. 1B: After a brief latency the animals attempted to turn away as long as the stimulus was presented. The strength of the avoidance response was strongly dependent on the angular expansion velocity of the stimulus with a maximal response at a velocity of 340 deg/s (Fig. 1C). Objects moving towards a fly with a constant velocity induce not a constantly but exponentially increasing expansion pattern on the retina. To mimic a physically realistic approach dynamic, we used looming squares and presented them with different patterns inducing either a decrease, an increase or no overall luminance change. A looming dark square (Fig. 1D), a bright square on a dark background (Fig. 1E) and a square with a checkerboard pattern (Fig. 1F) elicited similar avoidance responses independent of the global luminance change. In addition, dimming of a laterally presented square with 120° width induced even a slight turning towards the square (Fig. 1G). A looming horizontal bar expanding only vertically elicited an avoidance yaw turn (Fig. 1H) comparable in amplitude and time-course to the reaction away from a horizontally expanding bar. Finally, we replaced the expanding bar by two vertical bars moving away from each other for 0.25 s at a velocity of 360 deg/s. This elicited an avoidance behavior away from the stimulus (Fig. 1I). In summary, we found no or little influence of the overall luminance change on the reaction of the fly.

Avoidance turns could result from a different tuning of the optomotor response to front to back (FtB) and back to front (BtF) motion. To test this possibility we separated the looming bar stimulus, expanding in both directions, into single edge motion. When presenting a bar looming either FtB or BtF direction, a strong turning along with the respective edge direction was observed (Fig. 1J,K). However, when the bar was expanding in both directions, flies only turned along with the edge moving BtF, i.e. away from the stimulus. The sum of the responses to individual edges was clearly distinct from the response to the sum of both edges, i.e. the whole bar expansion (Fig. 1L).

**Fig. 2.**
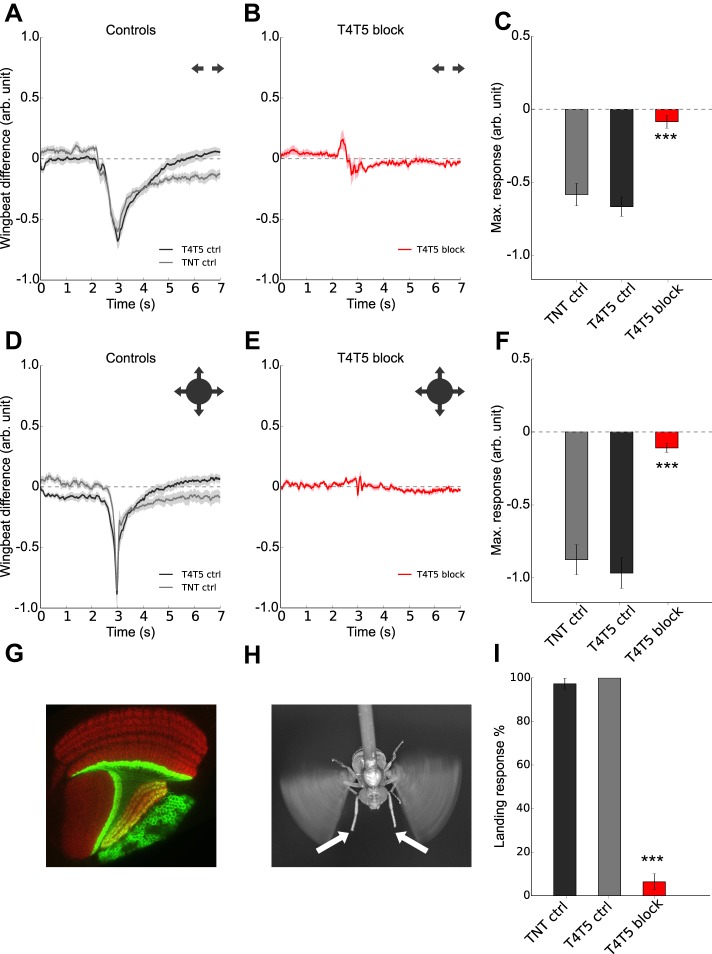
**T4 and T5 block abolished both landing and avoidance responses.** Flight behavior and landing responses of flies with TNT-E expression in T4 and T5 cells. (A) Turning responses of TNT and T4/T5 control flies to an expanding bar with an expansion velocity of 180 deg/s, *n*=12. (B) Turning responses of T4/T5 blocked flies to an expanding bar, *n*=12. (C) Maximal turning responses are significantly reduced in T4/T5 block flies (***P<0.001, two-sided *t-*test compared with both control groups). (D) Flight turning behavior of TNT and T4/T5 control flies in response to a looming circle, *n*=12. (E) Turning responses of T4/T5 blocked flies to a looming circle, *n*=14. (F) Maximal turning responses are significantly reduced in T4/T5 blocked flies (***P<0.001, two-sided *t-*test compared with both control groups). (G) GFP expression in T4 and T5 cells. (H) Example of a landing response. (I) Percentage of flies showing extension of their front legs in response to a looming square presented in front of them. TNT and T4/T5 controls showed a positive response in 97% and 100% of all trials, respectively, whereas T4/T5 blocked flies performed only 6.3% positive leg extension. This reduction was significant (***P<0.001, two-sided *t-*test compared with both control groups), *n*=11. All data represent mean±s.e.m.

Our data so far indicate that the avoidance behavior is distinct from the optomotor response, but depends on the evaluation of local motion signals rather than on overall luminance changes. Since T4 and T5 neurons are known to represent the elementary motion detectors in the fly brain (Maisak et al., 2013), we measured the avoidance behavior of flies with blocked T4/T5 cells. We silenced T4 and T5 cells by expressing the tetanus-toxin light chain (Sweeney et al., 1995) and measured the response of T4/T5 blocked flies to different looming stimuli. The response to an expanding bar was completely abolished in T4/T5 blocked flies (Fig. 2B) compared to both groups of parental control flies (Fig. 2A). To confirm this with another stimulus, we presented a looming circle, a stimulus eliciting very strong avoidance reactions in control flies (Fig. 2D). T4/T5 blocked flies did not react at all to this stimulus (Fig. 2E).

If presented in front of the fly, looming or expanding stimuli do not elicit avoidance turns, but rather a leg extension typical for the landing response (Tammero and Dickinson, 2002). We presented a looming square (expanding to 180° in 1 s) and captured images of the fly from above. We quantified the landing response as the percentage of positive front leg extension at the time point of expected collision with the square stimulus. Control flies almost always reacted with an extension of their front legs (TNT controls 97%, T4/T5 controls 100%), whereas T4/T5 blocked flies showed only 6.3% positive reactions (Fig. 2I). These data strongly indicate that the avoidance as well as the landing response are dependent on the activity of T4 and T5 cells.

Neurons reacting to looming stimuli and induce various kinds of avoidance or escape behaviors have been described in many animal models like locusts, crabs, pigeons and mice (Gabbiani et al., 1999; Oliva and Tomsic, 2014; Wang and Frost, 1992; Zhao et al., 2014). The detection of a looming stimulus can be realized in different ways. The giant fiber of *Drosophila*, a large neuron receiving part of its input from the lobula, elicits fast escape jumps (von Reyn et al., 2014) and reacts to approaching stimuli, sudden light-ON or light-OFF stimuli and mechanical stimulation (Mu et al., 2014). A giant lobula neuron in the locust, called LGMD neuron, is selectively sensitive to looming stimuli (Gabbiani et al., 1999). The angular size of a looming stimulus increases exponentially, which decreases the latency of the photoreceptor inputs. The LGMD synchronizes these excitatory inputs derived from progressing edges due to the successive latency decrease (Jones and Gabbiani, 2010). A different computation is used by PV-5, an approach-sensitive retinal ganglion cell of the mouse. PV-5 integrates excitatory OFF and inhibitory ON inputs which tunes the neurons to dark approaching or dimming objects (Münch et al., 2009).

In contrast, the landing and avoidance responses of flies were proposed to rely on summation of elementary motion detectors (Borst and Bahde, 1988; Tammero and Dickinson, 2002). We found that both behaviors are indeed dependent on the activity of T4 and T5 neurons, which become directionally selective by performing a spatiotemporal correlation of their input (Maisak et al., 2013), a computation described by the Hassenstein-Reichardt detector model (Hassenstein and Reichardt, 1956). Accordingly, in our experiments the avoidance response was elicited by the diverging edge motion of expanding or looming stimuli, independent of an overall luminance change or edge acceleration. T4/T5 neurons are grouped in four subtypes each tuned to motion in one out of the four cardinal directions. These T4/T5 subtypes project their axonal terminals into four adjacent layers of the lobula plate, where they form excitatory synapses onto the dendrites of lobula plate tangential cells (Maisak et al., 2013; Mauss et al., 2014). Our data suggest that an approach-sensitive neuron should receive excitatory input from T4/T5 cells in at least two lobula plate layers. Such a neuron would be activated by simultaneous activation of the two vertical or the two horizontal layers. There are cells in the flies optic lobe reported to be looming sensitive and influence escape behavior, the foma-1 neurons (De Vries and Clandinin, 2012). One of them has a dendrite located in the lobula plate and could be a candidate neuron for the avoidance and landing response.

Different visual behaviors use neural modules in the visual lobe which partially overlap with each other. In case of behaviors driven by expansion flow-fields, our results indicate that they share the circuits for elementary motion detection, i.e. T4 and T5 cells and their presynaptic circuitry, with the optomotor response and bifurcate at the level of large-field tangential cells of the lobula plate (Borst, 2014).

## MATERIALS AND METHODS

### Fly strains

Flies were raised on standard cornmeal-agar medium at 25°C and 60% humidity on a 12 h light/12 h dark cycle. The genotypes used are the following: Wildtype Canton-S flies, T4T5 block flies (w+/w−;UAS-TNT-E/R59E08-AD; R42F06-DBD/+), T4T5 control flies (w+/w−;R59E08-AD/+;R42F06-DBD/+) and TNT-E control flies (w+/w−;UAS-TNT-E/+,+/+). The T4T5 split Gal-4 line was kindly provided by Aljoscha Nern, HHMI Janelia Research Campus (GMRSS00324), the UAS-TNT-E flies derived from the Bloomington Stock Center (stock no. 28837).

### Behavioral experiments

We used female flies two days after eclosion. They were anesthetized by cooling to 3°C, glued to a needle with blue-light-activated cement with their heads fixed and, after recovery, placed into the arena. Visual stimulation was provided by three LCD screens arranged around the fly, controlled by a NVIDIA 3D Vision Surround Technology (Bahl et al., 2013). The fly turning behavior was measured with a ‘wingbeat analyzer’ (Gotz, 1987). Above the fly a camera (Grasshopper 03K2M+Infinity InfiniStix 94 nm/1.00×) helped to position it and allowed video tracking. Landing responses were measured as front leg extension.

### Data analysis and presented stimuli

Wing beat data were converted with an analog-digital converter from National Instruments (USB-6009). The left–right wingbeat signal difference was used as a value proportional to the yaw torque of the fly. The stimuli were presented at ±50° lateral to the flies with a contrast of 50% for wild type flies and 33% for T4/T5 blocked experiments. Each fly performed eight trials; trials and both sides were averaged to a mean turning response.
